# Potential Risks to Hearing Functions of Service Members From Exposure to Jet Fuels

**DOI:** 10.1044/2021_AJA-20-00226

**Published:** 2021-08-18

**Authors:** Thais C. Morata, Michelle Hungerford, Dawn Konrad-Martin

**Affiliations:** a National Institute for Occupational Safety and Health, Cincinnati, OH; b National Center for Rehabilitative Auditory Research, VA Portland Health Care System, OR

## Abstract

**Purpose:**

Several military occupations, particularly those within the U.S. Air Force, require working with or around jet fuels. Jet fuels contain components that are known to affect central nervous function, yet effects of these fuels on auditory function, specifically auditory processing of sound, are not well understood at this time. Animal studies have demonstrated that exposure to jet fuels prior to noise exposure can exacerbate the noise exposure’s effects, and service members exposed to jet fuels are at risk of noise exposure within their work environments. The purpose of this article was to give a brief synopsis of the evidence on the ototoxic effects due to jet fuel exposure to aid audiologists in their decision making when providing care for populations who are occupationally exposed to fuels or while during military service.

**Conclusions:**

Exposure to jet fuels impacts central nervous function and, in combination with noise exposure, may have detrimental auditory effects that research has yet to fully explain. Additional longitudinal research is needed to explain the relationships, which have clinical implications for service members and others exposed to jet fuels. In the meantime, audiologists can gain useful information by screening for chemical exposures when obtaining patient case histories. If jet fuel exposure is suspected, the Lifetime Exposure to Noise and Solvents Questionnaire can be used to estimate a noise exposure ranking and identify other potentiating agents such as jet fuel and industrial chemicals. A history of jet fuel exposure should inform the selection of hearing tests in the audiometric evaluation and when devising the treatment plan.

A large body of literature documents ototoxic effects of chemicals such as solvents, fuels, metals, asphyxiants, and pesticides following exposures in the workplace. These effects have been assessed by comparing rates of hearing loss between groups exposed to chemicals and nonexposed controls (e.g., Guest et al., 2012; Hughes & Hunting, 2013; Kaufman et al., 2005; Konopka et al., 2014; Morata et al., 1997; Prasher et al.,2005; Roggia et al., 2019). Group differences have also been noted in other types of auditory function, such as a decreased ability to understand speech, especially in the presence of background noise (using batteries of hearing tests designed to evaluate central auditory functions). The European Agency for Safety and Health at Work and the Nordic Expert Group have published comprehensive evaluations of ototoxic substances and have documented (a) disorders associated with workplace exposure to noise and ototoxic chemical substances, including qualitative information on noise-chemical interactions, and (b) key policies from specific countries and multinational conglomerates (European Agency for Safety and Health at Work, 2009; Johnson & Morata, 2010). In 2018, the U.S. Occupational Safety and Health Administration (OSHA) and the National institute for Occupational Safety and Health (NIOSH) published a safety and health informational bulletin to alert the occupational safety and health community of the potential risks from these exposures (OSHA & NIOSH, 2018). In 2019, the American Conference of Governmental Industrial Hygienists adopted an ototoxicant notation to highlight a chemical’s ability to cause hearing loss either alone or in combination with noise. If ototoxicant chemicals are present, the American Conference of Governmental Industrial Hygienists suggests noise exposure reduction using engineering, administrative, and personal protective equipment controls. They also suggest placing affected employees in hearing conservation and medical surveillance programs to monitor hearing thresholds. In this article, we aim to highlight the implications of the evidence on the ototoxicity of fuels to audiology practice.

Jet fuels, including jet propulsion fuel-8 (JP-8), like other hydrocarbon fuels, have components that are known to affect some hearing functions (Johnson & Morata, 2010; Mattie et al., 1995; Mattie & Sterner, 2011; Roggia et al., 2019). In particular, it is clear from animal models that exposure to hydrocarbon fuels, including jet fuels, can damage central nervous system function and thus could impair auditory processing of sound (Fechter et al., 2007, 2010, 2012; Guthrie et al., 2016). Several studies have reported an association between exposure to jet fuels and hearing outcomes; however, the exact conditions necessary for jet fuel exposure to affect hearing functions in humans have not been determined, and it is also unknown how long one would have to be exposed to be at risk (Fuente et al., 2019; Guest et al., 2012; Kaufman et al., 2005; Prasher et al., 2005; Wilson et al., 2010).

In animal studies, jet fuels alone do not appear to cause hearing loss; however, combined exposure to jet fuels and noise is another story (Fechter et al., 2007, 2010, 2012; Guthrie et al., 2014, 2015, 2016). When these exposures co-occur, jet fuels have been shown to enhance the damaging effects of noise on the auditory system. This increases the risk for noise-related hearing loss beyond the risk posed by the noise alone (Guthrie et al., 2016; Johnson & Morata, 2010; OSHA & NIOSH, 2018; Warner et al., 2015). Several mechanisms have been hypothesized for this effect. Some aromatic solvents appear to reduce the protective role played by the middle ear acoustic reflex (Campo et al., 2007; Wathier et al. 2019). A dysfunction of this reflex increases risks to hearing by allowing higher acoustic energy levels to penetrate the inner ear. Additionally, studies in animals suggest that the damage to the ear from jet fuels might not be detectable in an audiogram as hearing loss but could nonetheless cause neural damage that leads to secondary changes as the central auditory system adapts to a reduced peripheral input to the brainstem (Guthrie et al., 2014). Guthrie proposes that the central auditory processing disorder associated with this mechanism could impair brainstem encoding of stimulus intensity. This finding was present with chemical exposure alone, without concomittant noise exposure.

At this point, few studies have documented these central auditory effects in humans (Chen et al., 1992; Fuente et al., 2019; Odkvist et al., 1987; Prasher et al., 2005), and the existing information on the functional result of such an injury is incomplete. Audiologists and other health care providers would be wise to look beyond basic audiometric findings and incorporate self-reported hearing difficulties as an indication of possible central involvement (Guthrie et al., 2014). So far, the limited human studies in this area differ in their study designs and findings. Some studies suggest that jet fuels harm hearing, while other studies do not show a detrimental effect on hearing. Certainly, information on the exposure itself must be carefully considered when examining specific cases; however, research in this area suffers from a lack of detailed information on fuel exposure histories and any co-exposure with noise among the study participants. Noise exposure is common among those with exposure to jet fuels and can be considerable. In that case, the effects of noise are often more easily detectable than the effects of chemical substances (Hughes & Hunting, 2013; Konopka et al., 2014). Future efforts are encouraged to seek detailed exposure history and assessment of exposure to noise, jet fuels, and other ototoxicants.

The minimum time necessary for exposure to fuels to affect hearing has not been studied sufficiently, and defining the nature of the toxic process, whether acute or chronic, has not been possible (Johnson & Morata, 2010). Although more research would be needed to definitively answer this question, evidence suggests that—similar to noise exposure—increased duration of jet fuel exposure increases the odds of incurring hearing loss (Kaufman et al., 2005).

The toxicity of jet fuels also varies according to their components. JP-8, which is commonly used in military jets, is a kerosene-based fuel that contains several components that could harm hearing. That is why, while JP-8 is known to be less toxic overall than other types of fuel (Mattie & Sterner, 2011; Mattie et al., 1995; National Research Council Subcommittee on Jet-Propulsion Fuel 8, 2003), military and occupational exposure to JP-8 is evaluated and managed to be kept under control as a general principle.

## Types of Auditory Symptoms That Might Result From Jet Fuel Exposure

Ototoxic damage to the ear can cause hearing difficulties that may be temporary or permanent and can affect the ability to detect soft sounds. Because the sensory changes that limit sound sensitivity also broaden auditory filters and because chemicals may affect additional portions of the auditory system such as the neuronal pathways to the brain or the brain itself, sounds may not only need to be louder to be detected but may also not sound as clear as before (Warner et al., 2015). In particular, difficulties understanding speech in background noise may occur and involve changes in auditory processing such as:

- compressed loudness: sound distortion;
- poor frequency resolution: the inability to differentiate two sounds close in frequency;
- poor temporal resolution: the inability to detect time gaps between sounds; and
- poor spatial resolution: the inability to localize sound.

The most significant challenge in establishing causation is that the type of effects of jet fuel on hearing and auditory processing are not specific. Many factors could explain the difficulties in understanding speech in background noise described above. For example, it is common for older adults to have high-frequency sensorineural hearing loss, and this loss alone can affect auditory processing, making it harder to understand speech. In addition, older adults often experience mild cognitive changes as part of “healthy aging” that can contribute to auditory processing and speech understanding problems (Golding et al., 2004; for a review, see Humes et al., 2012).

From an audiological testing and auditory rehabilitation standpoint, it remains useful to obtain a detailed exposure history to help rule out certain disorders and determine whether auditory processing is impaired. See the report by Griest-Hines et al. (2021), which describes results of the Lifetime Exposure to Noise and Solvents Questionnaire that can be used to estimate a noise exposure ranking from low to high and identify other potentiating agents such as jet fuel and industrial chemicals. As audiologists are already aware, the pure-tone audiogram does not give a full picture of a patient’s hearing difficulties. When exposure to industrial chemicals is documented, audiologists have been encouraged to consider central auditory processing abilities in addition to severity of hearing loss to provide appropriate and comprehensive care (Fuente & McPherson, 2006; Morata & Little, 2002). In this article, we support that recommendation for jet fuel exposure as well. In addition to hearing aids, patients with ototoxic exposures may need technology to increase the signal-to-noise ratio or additional auditory rehabilitation services in order to have successful resolution to their communication barriers.

It should be noted that balance difficulties have also been associated with hydrocarbon exposure in humans, but few studies have specifically examined this effect for jet fuel and its numerous hydrocarbon components on the parts of the ear and brain responsible for balance control. The following studies suggest an association between impaired balance unidentified jet fuels (Odkvist et al., 1987) or JP-8 exposures (Fife et al., 2018; Maule et al., 2013; L. B. Smith et al., 1997).

## The Bottom Line About Risk and Some Caveats

While jet fuels’ effects on hearing have not been clearly established in exposed populations, the potential risk for an individual exists, particularly for increased difficulty with understanding speech in noisy environments and with other complex listening tasks. Taken together, the research findings mentioned above suggest that exposure to jet fuels could contribute to auditory processing problems and/or exacerbate noise-induced hearing loss. Hearing conservation efforts should continue to be promoted that include safe handling of hydrocarbon fuels, the provision of personal protective devices (including but not restricted to hearing protection devices and training on their use), and routine auditory evaluations of individuals who are exposed to hydrocarbon fuels and noise. Central auditory processing tests should be included as part of the test battery for evaluating possible effects of both jet fuel and noise.

Certain military occupations or duties may place individuals at risk for exposure to jet fuels. Within the U.S. Air Force, these may include refueling; entering the fuel tank; working in the aircraft maintenance group; handling fuel systems; working in the fuel handling group; mechanics; and flight line personnel, especially crew chiefs (Merchant-Borna et al., 2012; K. W. Smith et al., 2010, 2012). Individuals in other military branches working in and around aircraft could be similarly exposed. Civilian aviation occupations also may encounter the same types of hazards, though the extent of these hazards needs further study. Military personnel are routinely screened for changing health conditions, whereas employee health examinations are not as standardized or regulated (Karaanikas et al., 2021).

Similar concerns exist about other types of fuel and industrial solvents with hydrocarbon components (e.g., benzene, toluene, xylene, hexane, and heptane). These are used in the military and in nonmilitary occupational settings (Fuente et al., 2019; Johnson & Morata, 2010; Prasher et al., 2005). Auditory disorders have been reported to be more prevalent in workers from oil refineries (De Barba et al., 2005; Morata et al., 1997) and gas station attendants, when compared to nonexposed workers (Roggia et al., 2019).

OSHA and NIOSH (2018) identified as a first step in preventing exposure to ototoxicants is to know if they are in the workplace. When there is no information on a certain chemical’s ototoxicity, information on the chemical’s general toxicity, nephrotoxicity, and neurotoxicity may provide clues about the potential ototoxicity. Most chemicals that are known to affect the auditory system are also neurotoxic (Odkvist et al., 1987) and/or nephrotoxic, which can promote the release of reactive oxygen species (Abouee-Mehrizi et al., 2020; Ritchie et al., 2003). For example, synchronous noise and toluene exposure can affect the oxidant and antioxidant properties of kidney tissue by increasing plasma glutathione peroxidase, malondialdehyde dismutase activity, and superoxide dismutase (Abouee-Mehrizi et al., 2020). These same mechanisms may be involved in the biochemical processes through which jet fuel exposure impacts hearing or brain functions. In performing a risk assessment of work tasks, employers can evaluate the harmful effects of chemicals on their jobsites and make an informed decision as to whether or not a safer alternative is available. Work processes can be revised to eliminate the ototoxic chemical exposure altogether. If this is not feasible, engineering and/or administrative controls, such as reducing the number of exposed workers or reducing the duration of exposure, should be carefully considered.

## Diagnosing the Auditory Disorders That Might Be Associated With Exposure to Ototoxicants

Many factors throughout one’s life can influence so-called “age-related hearing loss,” and noise and chemicals are some of the factors that can lead to poorer hearing in old age (Campo et al., 2011). At this point, there is no reason to expect delayed hearing difficulties once exposure has ended (Hughes & Hunting, 2013). Although many people experience a decrease in hearing acuity with age, others do not, and it is not possible to predict who will and who will not develop hearing loss as they get older. The median hearing loss attributable to aging for a given age group cannot be generalized to all individuals in that age group (Flamme et al., 2020).

In a large cross-sectional study, Wilson et al. (2010) found that hearing thresholds, speech understanding in quiet and in noise, and self-perceived hearing handicap all became worse with age. There were no significant differences between Veterans and non-Veterans in this age effect. The study was not longitudinal, and it could not differentiate participants by their noise or jet fuel exposure history. Longitudinal studies are needed that evaluate hearing and central auditory changes among active duty military service members and veterans with different amounts of noise damage and jet fuel exposure in their careers. Research on the effects of noise and its interaction with other exposures including jet fuel is ongoing (Fuente et al., 2019). This includes experimental studies in animals, epidemiological studies comparing rates of hearing disorders among large groups with different exposure conditions, controlling for other effect modifiers and confounders, and in-depth longitudinal clinical studies with evaluation of central auditory function.

## Conclusions

The effects of hydrocarbon fuels, including jet fuels, on the auditory and vestibular systems are not completely understood at this time. Research in animal models has demonstrated that exposure to hydrocarbon fuels impairs central nervous system function and can increase the damaging effects of noise on hearing and cochlear afferent function, which are not detectable by an audiogram. The implications of this research are not well defined for humans; exact conditions needed for jet fuel exposure to affect hearing function are unknown. Although necessary research is lacking, the current research does suggest that exposure to jet fuels could contribute to auditory processing problems and exacerbate noise-induced hearing loss.

Although the current research landscape is incomplete, audiologists can take action now to provide enhanced care to their patients exposed to jet fuels. Self-reported hearing difficulties that are not consistent with pure tone audiometric results can indicate the need for further hearing testing to complement the assessment. Audiologists can seek to obtain more information on their patients’ exposure to hydrocarbon fuels. Any patients at risk for exposure can complete a questionnaire, such as the Lifetime Exposure to Noise and Solvents Questionnaire, to aid the audiologist in further clinical decision making, including when to complete additional test batteries. If the majority of audiologists would screen for chemical exposures, they could ensure patient care needs are fully assessed.
